# Capacitive-resistive radiofrequency therapy to treat postpartum perineal pain: A randomized study

**DOI:** 10.1371/journal.pone.0231869

**Published:** 2020-04-27

**Authors:** Florence Bretelle, Chantal Fabre, Marine Golka, Vanessa Pauly, Brimbelle Roth, Valérie Bechadergue, Julie Blanc

**Affiliations:** 1 Department of Gynecology and Obstetrics, AP-HM, Assistance Publique-Hôpitaux de Marseille, Marseille, France; 2 Unité de Recherche sur les Maladies Infectieuses Tropicales et Emergentes, UM63, CNRS 7278, IRD 198, INSERM 1095, Marseille, France; 3 Midwife school, Faculty of medical and paramedical sciences, Aix-Marseille University, Marseille, France; 4 Medical Evaluation, Department of Public Health, Assistance Publique-Hôpitaux de Marseille, AMU, Aix- Marseille Université, Marseille, France; 5 EA 3279, CEReSS, Health Service Research and Quality of Life Center, Aix-Marseille University, Marseille, France; Poissy-Saint Germain Hospital/Versailles Saint Quentin University, FRANCE

## Abstract

**Objective:**

To evaluate the reduction of perineal pain after vaginal deliveries by capacitive resistive radiofrequency therapy (RF).

**Methods:**

We conducted a double-blind randomized study in University Hospital Centre in France. We included women presenting either perineal tears or an episiotomy after vaginal delivery (instrumental assisted or not). The participants were randomly assigned to RF or not at day 1 and day 2 postpartum. The primary outcome was pain evaluated as visual analog scale (VAS) score >4 at rest on day 2 after the treatment. Secondary outcomes included discomfort and pain while walking and seating two days after treatment, type of pain two days after treatment and analgesics intake two days after treatment, sexual intercourse retake and painful of intercourse were also assessed by phone call 30 days after delivery. We performed univariate analysis and multivariable regressions adjusting on the value of the outcome at baseline to improve precision of the estimated intervention effect.

**Results:**

Between June 1, 2017 and October 8, 2017, the RF group included 29 women compared with 31 women in the group without RF. There was no significant difference on VAS >4 between the two groups (13.8% vs. 9.7% p = 0.69; difference = 4.1%, 95%CI -12.2%- 20.4%); consumption of paracetamol was lower in the RF group (978.3 mg (sd = 804.5) vs 1703.7 mg (sd = 1381.6), p = 0.035; difference = -725.3 mg, 95%CI -1359.6 - -91.3). Multivariate analysis showed no association between RF and pain. Nevertheless, we found an association between RF and discomfort while walking (adjusted OR 0.24, 95% CI 0.07–0.90; p = 0.03).

**Conclusion:**

VAS>4 at day 2 was not different in the experimental and the control groups but RF was associated with less perineal discomfort while walking and lower consumption of paracetamol after delivery.

**Clinical trial registrations:**

The study was registered in the Clinical Government trial (https://clinicaltrials.gov/ct2/show/NCT03172286?term=bretelle&rank=2) under the number NCT03172286.

## Introduction

The majority of women who have vaginal deliveries experience perineal pain 24h after birth (95%) [1]. This reaches 100% in the event of perineal tears. This pain lessens over the days following the birth but at 7 days after birth 60% of women with an intact perineum and 91% of those with perineal tears still suffered from incapacitating pain [1,2]. Progression towards chronic pain concerned almost 13% of women who had an episiotomy [3]. Step one analgesics (paracetamol in particular) are of variable efficacy [4,5]. Non-steroidal anti-inflammatory (NSAI) drugs are frequently used as they seem to efficiently relieve this type of pain [6,7]. Non pharmacologic therapies have to be developed because of breastfeeding in majority of women and risks of potential adverse effects of pharmacologic treatments [8]. Alternative techniques are currently under development such as perineal infiltration with local analgesics prior to perineal suturing [9,10] or the injection of hyaluronidase during labor [11].

Radiofrequency (RF) or high frequency therapy is commonly used in sport [12–14], low back pain but also in traumatology and urology [15–18]. It is used to provide rapid pain relief for sport injuries and allows quicker recovery [12–14]. Tecar therapy (TECAR: Capacitive and Resistive Energy Transfer) is an endogenous thermotherapy, which consists in the emission of high frequency waves via an applicator. The transfer of electromagnetic energy stimulates targeted tissues. RF can be used in two ways: capacitive or resistive. The capacitive mode (CET) concentrates the energy to target soft tissues containing electrolytes: muscles, vascular or lymphatic tissues. The resistive mode (RET) targets denser tissues containing more fat and fiber (such as bones, ligaments and tendons). High frequency waves penetrate deep into tissues and increase both exchanges and temperature and a recent study has demonstrated the effects of RF on the skin microcirculation and the intramuscular blood flow [19,20]. This has been shown to increase vascularization and reduce inflammation and swelling and to accelerate the healing process and provide pain relief [12]. To date, no study has evaluated the impact of RF treatment on postpartum perineal pain.

We hypothesized that capacitive and resistive radiofrequency therapy applied on perineal tears would decrease pain and improve mother well-being during post-partum period. The main objective was to assess the efficacy of radiofrequency therapy on perineal pain in postpartum for women presenting perineal tears. The secondary objectives were to assess discomfort and pain while walking and seating, type of pain and analgesics intake, and sexual intercourse retake and painful of intercourse. We aim to propose an innovative alternative approach in caring for women with post-partum tears and would achieve this aim by a double-blind randomized study.

## Materials and methods

We performed a double-blind single-center randomized controlled trial carried out in the University Hospital Centre North Marseilles, France.

The participation in the study was proposed to women after delivery in the postpartum hospitalization room.

Inclusion criteria were: primiparous or multiparous women presenting perineal tears (perineal tears of at least grade 2 or an episiotomy) after vaginal delivery, instrumentally assisted (vacuum device, spatulas, forceps) or not.

After having signed an informed consent form, women were randomized into two groups (with or without RF) via a randomization list. Randomization used a permuted block design (1:1 ratio).

In both groups the treatment sessions lasted for 15 minutes and were carried out on days 1 and 2 postpartum in a supine position with legs bent by the midwife. The flat transducer was applied on the perineum and specifically on the injury. Latex cover protected the flat transducer.

The judgment criterion endpoint was the percentage of woman with a VAS>4 on the numeric analogic scale on the second day postpartum after the treatment. VAS is a 10-centimeter horizontal continuous scale anchored from no pain (score of 0) to the worst imaginable pain (score of 10). The threshold of 4 was chosen because its clinical and therapeutic relevance and as already published by our team [9]. Secondary outcomes were prespecified: discomfort and pain while walking and seating (yes/no) on the second day after the treatment, type of pain (burning, tightness, shooting, stabbing, pricking) two days after treatment and measured as binary variables; and total paracetamol intake two days after treatment in milligrams.

Clinical and obstetric data were collected (age, parity, labor length, pushing time, spontaneous delivery, episiotomy), as well as data concerning pain: presence of hematoma (yes/no), pain while seating and walking (yes/no), discomfort while walking (yes/no), edema (yes/no). Pain was evaluated at rest when lying in bed using visual analog scale (VAS) before and after each session (at day 1 and day 2). A qualitative evaluation of pain was realized with using a VAS for each type of pain: burning, tightness, shooting, stabbing, pricking. Sexual intercourse retake and painful of intercourse were assessed by phone call 30 days after delivery. No recommendation was typically made to women about resumption of intercourse. Analgesic intake was measured; treatment with analgesics may include up to 3g/day of paracetamol, up to 300mg/day of ketoprofen or opioids if necessary. As, after delivery women may have benefited from perineal infiltration with ropivacaine 10 ml before perineal suture, this information has been collected.

MG entered the database and the funder AP-HM validated the accuracy of the database.

The medical device used was a BACK1S. Data analysis was totally independent of Winback and carried out by FB, BR and VP. The device is a high frequency therapy device, registered and exclusively commercialized by Winback, France and produced by Daeyang, South Korea in compliance with 93/42/EEC under the number 1984-MDD-14- 108 314.

The study received a favorable opinion from the ethics committee approval from the French Obstetrics and Gynecological Research Ethics Committee (CPP) on October 12, 2016 and was registered under number 1697 and ID RCB: 2016-A01499-42. It was registered in the Clinical Government trial ClinicalTrials.gov Identifier: NCT03172286. The study was recorded in the register of the Institutional Review Board, the French data protection commission CIL/AP-HM under number 2018–40.

### Group A: Radiofrequency therapy

Women received radiofrequency therapy using a BACK 1S device emitting radiofrequency waves. The Back1S device used for the clinical study has 3 frequencies: 300KHz, 500KHz and 1MHz. Each frequency is supposed to allows targeting the depth of action: the lower the frequency, the deeper is the action. During this study two frequencies were used (300 and 500KHz). The capacitive (CET) mode is an application mode that has a superficial action (2 to 3 cm) on soft tissues (high water content). The resistive (RET) is an application mode that allows crossing the overall depth of tissues and acts on all fibrous tissues (low water content). The dynamic function used within this protocol allows the device to switch automatically from frequency 300KHZ to 500KHz every 3 seconds. The device was used with power of 12 Watts. The treatment time was 15 minutes two times. Among these 15 minutes, the first phase of the protocol included a CET phase of 5 minutes. During this phase two static electrodes of 13,5 cm^2^ each were applied on the sacrum and on the pubis. After this first phase, a RET phase was applied for 10 minutes using a 35 mm diameter flat applicator emitting high frequency waves. This applicator was applied in direct contact with the perineum and specifically on the injury.

### Group B: Without radiofrequency therapy

Upon inclusion in group B, women were treated by applying the same protocol with firstly the static electrodes for 5 minutes (on the sacrum and the pubis) then the same flat transducer was applied in direct contact with the perineum for 10 minutes without emitting any waves in order to respect the blind aspect of the study. The overall treatment time was the same as for group A: 15 minutes.

In both groups, the machine didn’t emit sounds and the screen of the machine was hidden in order not to aware women about randomization group.

The midwife who carried out the radiofrequency therapy had to follow a strict and uniform protocol in each group. A different midwife, who had no knowledge of the randomization group, evaluated each woman was by using the numeric analogic scale before and after each session, at day 1 and day 2.

The sample size required to achieve a power of 1-β = 0.90 with a α level equal to 0.05 to detect a difference of 40% of women with VAS>4 between groups (80% in the No RF group [1] vs 40% in the RF group) at day 2, was 60 women in total (PROC POWER SAS® for the two sided chi-square test). The difference of 40% was chosen because more realistic than scarce previous study (more than 70%) [18] and taking into account the current protocol of infiltration with ropivacaine in case of perineal tears. We provided to include 32 women in each group in case of lost of follow up.

Groups were compared at baseline using Chi square statistical test (or Fisher’s exact test) for qualitative data and Student’s t-test (or non parametric Kruskall Wallis test) for quantitative data. Categorical variables were given as the number of observations and percentages; quantitative variables were given as mean and standard deviation (mean+/-SD).

We compared the groups after treatment at day 2 on the following outcomes (VAS>4, quantitative VAS, pain and discomfort while walking and seating, using logistic (for binary outcomes) and linear (for quantitative ones) regressions. First, we performed univariate regressions (logistic or linear depending of the nature of the outcome) and we secondly performed multivariable regressions adjusting on the value of the outcome at baseline to take into account imbalanced groups at baseline despite of the randomization. We analyzed and compared paracetamol intake between groups on the second day before the 2^nd^ session by performing a univariate and a multivariable analysis of covariance of the quantities of paracetamol, taking into account baseline consumption of paracetamol and the group.

## Results

**Fig 1:**
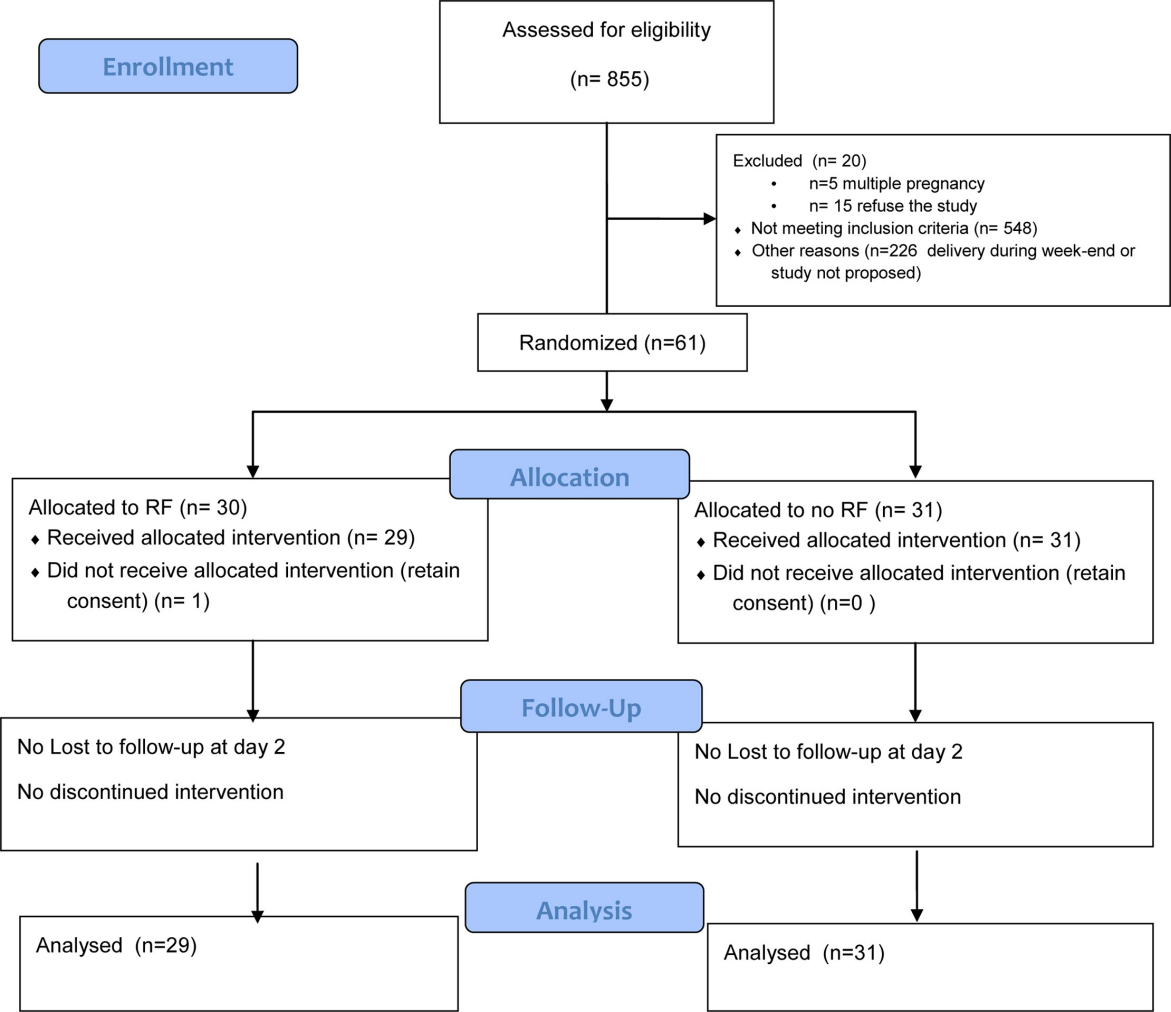
CONSORT diagram for study participation.

Between June 1, 2017 and October 8, 2017, 855 women gave birth and 212 of them by caesarean section. The flow chart is shown in Fig 1.

Sixty-one women were enrolled in the study and randomized into either the group A (n = 30) receiving RF therapy or the group B without RF (n = 31) during the pre-defined inclusion period. One woman after having given her informed consent and been randomized into the RF group A did not wish to continue and was excluded from the study, thus the RF group finally included 29 women. There was no woman lost to follow up on the second day (day of main outcome measurement). At day 30, the number of lost to follow up was 10 in the group A (33%) and 8 in the group B (25.8%).

Clinical characteristics of women are summarized in Table 1. The women of Group A with RF presented significantly more edema than group B and higher but non-significant longer length of labor as well as pushing time (Table 1). Baseline measures of discomfort and pain demonstrated higher percentages of women presenting pain and discomfort while walking and pain while seating in group A.

**Table 1:** Baseline comparisons and pain evaluation between groups (univariate analysis) with or without RadioFrequency (RF).

Characteristics	Group A	Group B	*p*
	With RF	Without RF	
	N = 29	N = 31	
Age (y), mean (SD)	26.6 (4.5)	28.8 (6.3)	0.13
Parity, mean (SD)	1.5 (1.1)	1.9 (1.1)	0.13
Labor length (min), mean (SD)	362 (225)	261 (208)	0.05
Pushing time (min), mean (SD)	18.6 (13.3)	14.4 (14.0)	0.10
Spontaneous delivery^a^, n (%)	23 (79.3)	29 (93.5)	0.14
Hematoma^a^, n (%)	4 (13.8)	0 (0)	0.05
Edema^a^, n (%)	16 (55.2)	8 (25.8)	0.02
Episiotomy^a^, n (%)	5 (17.2)	3 (9.7)	0.46
Perineal Infiltration with Ropivacaine^a^, n (%)	7 (24)	4 (12.9)	0.22
Pain while walking^a^, Day 1, n (%)	24 (82.8)	19 (61.3)	0.03
Discomfort while walking^a^, Day 1, n (%)	28 (96.6)	23 (74.2)	0.03
Pain while seating^a^, Day 1, n (%)	29 (100)	25 (80.6)	0.02
Discomfort while seating^a^, Day 1, n (%)	28 (96.6)	27 (87.1)	0.20
Consumption of Paracetamol^a^, n (%)	25 (86.2)	27 (87.1)	0.61

RF, Radiofrequency; VAS: Visual Analog Scale

^a^ Binary variables (yes vs no)

Data are mean (SD) or n (%)

At Day 2, the women of group A with RF didn’t present significantly more edema than group B before the second session of treatment (13.8% in group A vs 12.9% in group B, p = 0.99; difference = 0.90%, 95%CI -14.7%—;18.3%).

After univariate and multivariate analysis, no significant differences were observed between the groups regarding main judgment criteria: pain at rest on the second day after treatment evaluated by VAS (both VAS>4 criteria–Difference = 4.1%, 95%CI -12.2% - 20.4% (Table 2) and mean VAS criteria: Mean difference = 0.42; 95%CI -0.57–1.40 [not presented]) Multivariable analysis (adjusted on value at baseline) showed a statistical association between discomfort while walking (adjusted Odds Ratio [aOR] 0.24; 95%CI 0.07–0.90, p = 0.03) and a lower consumption of paracetamol in group A with RF (Adjusted difference = -642.0, 95%CI -20.7–1304.7, p = 0.05).

**Table 2:** Main and secondary judgment measures (Univariate and multivariable analysis).

Characteristics	Univariate analysis			Multivariable analysis^a^		
	With RF n = 29	Without RF n = 31	*p*	aOR / Beta coefficient	95% CI / Standard error	*p*
VAS >4 at rest, Day 2 after treatment; n (%)	4 (13.8)	3 (9.7)	0.69	1.91	0.36–10.09	0.45
Pain while walking Day 2 after treatment^b^ n (%)	11 (37.9)	11 (35.5)	0.67	0.67	0.20–2.30	0.53
Discomfort while walking Day 2 after treatment^b^ n (%)	12 (41.4)	20 (64.5)	0.08	**0.24**	**0.07–0.90**	**0.03**
Pain while seating, Day 2 after treatment^b^ n (%)	18 (62.1)	15 (48.4)	0.14	1.90	0.60–6.05	0.27
Discomfort while seating Day 2 after treatment^b^ n (%)	14 (48.3)	21 (67.7)	0.14	0.39	0.12–1.28	0.12
Total paracetamol intake two days after treatment (mg), mean (standard error-SE)	978.3 (167.8)	1703.7 (265.9)	**0.035**	-642.0	329.4	0.05

^a^ Multivariate regressions adjusting the value of the outcomes at baseline and group of treatment

^b^ Binary variables (yes vs no)

RF, Radiofrequency; aOR, adjusted Odds Ratio; 95% CI, 95% confidence interval; VAS, Visual Analog Scale

Data are n (%) or mean (SE)

Qualitative evaluation of pain showed similar characteristics between groups after treatment (Table 3).

**Table 3:** Secondary judgment measures: qualitative evaluation of pain at day 2 and sexual activity retake at day 30 (Univariate analysis).

Characteristics	With RF n = 29	Without RF n = 31	*p*
**Qualitative evaluation of pain (VAS), Day 2 after treatment, mean (SE)**			
**Burning**	2.00 (0.52)	1.20 (0.36)	0.29
**Tightness**	1.28 (0.43)	1.97 (0.48)	0.27
**Shooting**	2.14 (0.45)	1.90 (0.43)	0.74
**Stabbing**	0.97 (0.36)	0.87 (0.32)	0.89
**Pricking**	0.76 (0.36)	0.60 (0.27)	0.92
**Sexual activity retake Day 30^a^, n (%), n = 42**	9 (47.4)	8 (36.8)	0.41
**Painful sexual intercourse^a^, n (%), n = 42**	4 (21.1)	3 (13.0)	0.68

RF, Radiofrequency; VAS, Visual Analog Scale

^a^ Binary variables (yes vs no)

Data are mean (SE) or n (%).

On day 30 postpartum, 47.4% of women in the RF group and 36.8% in the group without RF reported having resumed sexual activity (p = 0.41). Sexual intercourse was reported as painful for 21.1% of women in the RF group and 13.0% in the group without RF (p = 0.68) (Table 3).

## Discussion

This randomized trial showed no superiority of the RF regarding perineal pain evaluated using a VAS on the second day after delivery. However, our study showed that women who were administered RF felt significantly better with less perineal discomfort when walking and consumed less paracetamol at day 2 after delivery.

To our knowledge this is the first study evaluating the impact of RF during the postpartum period. Our study did not perform to show less VAS >4, main judgment criteria in the RF group. There was no superiority of RF for pain improvement and possibly because of the small sample size, the result was not in favor of RF concerning the main judgment criteria. This could be linked with the absence of effectiveness of the technique, which is unlikely regarding its use in other painful situations [14–18]. This could also be linked to the intervention’s duration, which could be insufficient to measure the impact on perineal pain. However, our funding and staff constraints did not allow us to pursue RF sessions after hospital discharge. Nevertheless, scientific literature and recommendations regarding Tecar therapy efficiency are scarce [21–22]. Recently a paper reported a faster recovery among runners with greater increases in stride length, angle and height between two tests compared to a control group [12]. Moreover a small randomized study on low back pain reported benefits of Tecar therapy [15]. Nevertheless pain mechanisms may be different in the postpartum period compared with trauma and could explain discrepancies.

The absence of difference on the VAS scale at rest could be also linked with lack of power. Indeed, we hypothesized that 80% of women presented perineal pain after delivery and that RF would reduced this percentage to 40% while only 53.3% of women included presented VAS >4 at baseline (48.3% in group A with RF and 58.1% in group B without RF). This lower rate of women with perineal pain observed in our study may be linked to the use of perineal infiltration with ropivacaine prior to suturing which could strongly decrease the level of pain reported by women. Next studies should include only painful women to avoid this limit. Furthermore, it is necessary to evaluate on a larger scale to ensure even the safety of the technique in view of the results of this pilot study.

Despite randomization, the groups were not strictly identical. This can occur when the number of women involved in randomization is small. At baseline the RF group had more severe perineal tears with more frequent edema and a longer (but not significant) length of labor, which are well known as pain factors [23]. However, the women of group A with RF didn’t present significantly more edema than group B at the second evaluation (Day 2) before the second session of treatment. Because of randomization, it would have been unnecessary to make any adjustment; nevertheless due to imbalanced data on pain and discomfort values at baseline we have decided to adjust at least on these values so as to improve power in the analysis [24]. We chose to evaluate pain and discomfort even if they are related but not entirely as discomfort may be a sensation not associated with pain for example in case of edema [1,2].

We couldn’t perform a larger study because the promotion was defined for 124 RF treatment sessions maximum during a pre-defined period and with corresponding funded overtime hours for midwives. The limited funding also explained the high percentage of lost to follow up in each group (31% and 25.8% of women), 30 days after delivery, with follow up by phone call and not by scheduled consultation.

One of the limits of the study was the inclusion criterion that should be more precise for an objective assessment taking into account for example postpartum hemorrhage, breastfeeding and other elements that could be painful in postpartum period.

The decrease in medication use is a strong and objective indicator of efficacy compared with the VAS pain evaluation. Our results showed a reduction in the paracetamol cumulative dose in the RF group while the number of VAS >4 patients was higher in the RF group (but not significant). This paradoxical side could be explain by the characteristics of the pain, with more (but not significant) burning pain in the RF group. In case of burning pain, women may use less paracetamol but more local treatment like ice pads for example (data not available). A recent study showed that the extent of perineal pain postpartum led 28% of physicians to prescribe opioids [25]. However, in our practice, opioids are very rarely given except in cases of major vulvar hematoma. RF enabled paracetamol intake to be reduce which could decrease the prescription and probably the use of other analgesics. This consideration is essential because 66% of women in France and 81% of women in the United States initiate breastfeeding after delivery [8,26].

Moreover, after multivariate analysis the results of the present study showed that women had significantly less discomfort when walking. Even if this result could be due to a type I error, this is an interesting result for the woman and their relationship with their newborn baby. The assessment of pain during sexual encounters could be discussed and further studies should use more standardized sexuality questionnaires such as PISQ-12 [27].

The generalization of RF use in post-partum is not recommended in the light of our sole study, especially because of its size. However, the clinical relevance of these results suggests investigating the interest of RF in perineal symptoms after vaginal deliveries and with a more global evaluation of pain several days after delivery. A larger randomized study with more power is justified to investigate the efficacy of RF in immediate perineal pain after delivery and long-term pain.

## Conclusions

RF therapy had no significant impact on perineal pain at rest evaluated by a visual scale in this pilot study. RF treatment showed a significant reduction in perineal discomfort while walking and could halve the use of analgesics, which could improve well-being during this sensitive period.

## Supporting information

S1 Checklist CONSORT 2010 checklist of information to include when reporting a randomised trial*. (DOC)

S1 File (XLSX)

S2 File (DOCX)
