# Supplementary material for: Capacitive-resistive radiofrequency therapy to treat postpartum perineal pain: A randomized study
Source: PLoS One. 2020 Apr 27;15(4):e0231869. doi: 10.1371/journal.pone.0231869 (PMC7185583; doi:10.1371/journal.pone.0231869)
Supplement: S2 File — (DOCX) [file pone.0231869.s003.docx]

**CLINICAL  RESEARCH PROJECT**

**Impact OF RADIOFREQUENCY STIMULATION ON PERINEAL PAIN DURING POSTPARTUM.**

PRINCIPAL INVESTIGATOR

Pr Florence BRETELLE

Gynaecology Department

Hôpital Nord

Assistance Publique des Hôpitaux de Marseille

(Marseille Public Hospitals)

SPONSOR

Assistance Publique-Hôpitaux de Marseille

Direction de la Recherche Clinique et de l’Innovation

80, rue Brochier

13384 Marseille CEDEX 05

N°IDRCB : 2016-A01499-42

PROTOCOL

Version  n° : 2 of 07/11/2016


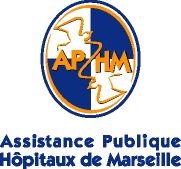


Table of Contents

1. **Background**
2. **Introduction**
3. **Research Objectives**
   1. Primary objective
   2. Secondary objective
4. **Methodology**
   1. Choice of experimental plan and justification
   2. Teams involved
   3. Study population
      - 1. Patient recruitment
        2. Selection criteria
           1. Inclusion criteria
           2. Non inclusion criteria
           3. Exclusion criteria
   4. Care groups
      - 1. Group A : Radiofrequency therapy
        2. Group B : No radiofrequency therapy
   5. Judgment criteria
      - 1. Primary judgment criterion
        2. Secondary judgment criteria
        3. Evaluation
   6. Planned number of patients and justification
   7. Duration of participation
5. **Diagram and course of the trial**
   1. Task distribution
   2. Monitoring organisation
6. **Data processing**
   1. Data collection
      - 1. Organisation
        2. Data collected upon inclusion
        3. Data collected during sessions
   2. Data entry
   3. Statistical analysis of data
      - 1. Analysis populations
        2. Description of the population, initial comparability between the groups
        3. Analysis of the primary judgment criterion
        4. Analysis of the secondary judgment criteria
   4. Serious adverse event management
7. **Planned duration of the trial**
8. **Expected results-Perspectives**
9. **Vigilance of clinical trials**
   1. Definition
      - 1. Adverse event
        2. Serious Adverse Event (SAE)
        3. Unexpected adverse events
   2. Investigator’s responsibilities
      - 1. Modalities for detection and collection of adverse events
        2. SAE reporting
        3. Evaluation of causality
        4. Quality control
10. **Legal and ethical aspects**
11. **References**
12. **Appendix: questionnaires**
13. **Background**

This study is within the framework of an end-of-study dissertation for a French state diploma in midwifery.

1. **Introduction**

The genital life of a woman goes through several main stages: maternity is one of them. It is a very important period and its consequences can perturb a young mother’s day-to-day life.

A study carried out on the postpartum period revealed that among the women who had undergone vaginal delivery and had perineal lesions, between 95 and 100% of them suffered from perineal pain (episiotomy, 1^st^ to 4^th^ degree tears) 24 hours after vaginal delivery; and 60 to 91% of them were still suffering 7 days after giving birth. ^[1]^ Another study on the incidence of perineal pain postpartum confirmed these figures. ^[2]^

Acute pain that goes untreated can become chronic pain; this concerns 12.8% of women who had an episiotomy. ^[3]^ This chronic pain is defined by the HAS (French Higher Health Authority) as pain lasting longer than usual, for which treatment is not sufficiently efficient and which can alter the functional and relational capacities of the patient. ^[4]^ According to the Association Québécoise de la Douleur Chronique (Quebec Association for Chronic Pain), this has physical and psychological consequences. It is therefore primordial to prevent this risk in young mothers since pain could prevent them from thriving in their new role.

On the postnatal ward, midwives are often at a loss with regards to pain relief for these patients. It is common practice to be told after birth: “it will go away”, unfortunately this is not always the case, and patients may continue to feel unwell for a long time because of it.

Only Step I analgesics are given (Doliprane^®^, Dafalgan^®^), which are not reliably efficient, with only a few studies proving their efficacy. ^[5, 6]^ They are frequently associated with non-steroidal anti-inflammatory drugs which seem to be efficient in treating this pain. ^[7, 8]^

Other techniques are being developed, including perineal infiltration five minutes before perineal suturing ^[9,10]^ or the injection of hyaluronidase during the active phase of labour. ^[11]^

Suturing techniques (individual interrupted sutures, continuous sutures, no sutures for minor lesions) are also known to play a non negligible role in perineal healing and pain. ^[12]^

Radiofrequency, which is an old technique, has already proven its worth in sport ^[13, 14]^, but also in traumatology ^[15, 16, 17, 18]^, urogynaecology ^[19, 20]^ and in beauty treatments. ^[21]^ It provides immediate and rapid pain relief for sport injuries and also enables rapid recovery and return to sport practice. It has also been shown to reduce oedema and bruising of hamstring injuries in footballers. ^[13, 14]^

The principle of radiofrequency, also known as Tecartherapy from the French acronym TECAR (Capacitive et Resistive Energy Transfer), consists in the emission of high frequency long waves (from 0.8MHz to 1.2MHz) applied using a flat or endocavity transducer. More precisely, it consists in the transfer of electromagnetic

energy at a given frequency in order to stimulate the same transfer within targeted tissues. Radiofrequency can be used in two ways : capacitive or resistive.

The capacitive mode concentrates energy via the transducer to target soft tissues containing electrolytes : muscles and vascular or lymphatic tissues. This increases exchanges within the tissues. ^[22]^

The resistive mode targets denser tissues, containing more fat and fibre (such as bones, ligaments and tendons). This mode penetrates deep into tissues and increases both exchanges and temperature. ^[13]^

Physically, these actions, combined with tissue resistance, cause an increase in temperature within the tissues in an endogenous way that does not generate contractures.

There are different types of generated effects: ^[14, 15, 23]^

- - - An increase in vascularisation due to thermal phenomena enabling to reduce inflammation and oedema and therefore pain
    - An increase in the speed of the natural healing process thanks to non-thermal processes. It modifies the electrochemical balance of the tissues and increases metabolic activity (ion exchanges, blood and nutriment supplies). This results in a quicker healing process and therefore reduced pain

1. **Research objectives**
   1. **Primary objective**

The primary objective of this trial is to evaluate the efficacy of radiofrequency treatment on perineal pain during postpartum, following vaginal delivery, instrumentally assisted or not, for patients with perineal lesions.

- 1. **Secondary objectives**

Secondary objectives are the following:

- - - To determine the impact of radiofrequency treatment on the patients’ quality of life and comfort during their stay on the maternity ward
    - To quantify the intake of analgesics during their stay
    - To quantify the length of time until sexual intercourse and intercourse quality during the postpartum period
    - To describe healing of the lesions

1. **Methodology**
   1. **Choice of experimental plan and justification**

A double-blind single-centre randomized prospective study will be carried out in the University Hospital Centre North Marseille, on patients hospitalised in the postnatal department.

Patient recruitment will be prospective. Two groups/strategies are described in detail in Chapter D of the methodology section of this project.

The patients will be randomized into two groups, one receiving radiofrequency therapy, the other with no radiofrequency therapy; using a randomization list.

The study will be double-blinded, only the person administering the treatment will know which group the patients belong to in the study. The assessor will not know which group the patients belong to in the study.

- 1. **Teams involved**
     - The midwives on the labour ward
     - The postnatal department of the North Hospital (AP-HM)
     - The public health hub of the AP-HM : V. PAULY
     - Winback Laboratories, WINBACK Europe, 855 Av du Docteur Lefebvre, Twins 2, 06270 Villeneuve-Loubet- France
     - The scientific committee: F. BRETELLE, V. BECHADERGUE, V. VAUGIRARD, A. CHARTIER, C. d’ERCOLE, C. FABRE
  2. **Study population**
     - 1. **Patient recruitment**

Women who have given birth at the North Hospital, who have perineal lesions (tears, episiotomy) will be offered to participate in this study.

All of the midwives on the labour ward or in the postnatal department, who have been informed of recruitment modalities (via an information sheet intended for midwives on the labour ward) will be able to include patients.

During inclusion by the midwife, after having checked the inclusion criteria and having the informed consent form signed, the midwife will collect data from the patient’s medical record and from her interview.

The patient will be randomized into one of 2 groups using a pre-established randomization list, after having signed the informed consent form:

- if the patient is included in group A: “radiofrequency therapy”

- if the patient is included in group B: “no radiofrequency therapy”

Randomization will be carried out by the midwife on duty.

- - - 1. **Selection criteria**
         1. **Inclusion criteria**
- Primiparous or multiparous patients with vaginal delivery
- Instrumentally assisted expulsion or not (vacuum device, spatulas, forceps)
- Presence of a perineal lesion (all degrees of tears, episiotomy)
- Women who understand the course and the objectives of the study and have signed an informed consent form.
  - - - 1. **Non inclusion criteria**
- Under-aged women (under 18 years old)
- Delivery by Caesarean section
- Presence of an intact perineum
- Women who have not signed the informed consent form
  - - - 1. **Exclusion criteria**
- Women who withdraw their consent during the study
  1. **Care groups**

The randomization list will be drawn up before the study begins. It will be designed under the responsibility of the Public Health Hub (Vanessa PAULY Hospital-Statistician Engineer, AP-HM).

The procedure upon each inclusion is the following: the midwife includes the patient after having collected necessary data then randomly assigns her to a care group (group A or group B) using a pre-established list.

- - - 1. **Group A: Radiofrequency therapy**

After inclusion in group A, patients will be treated using a WINBACK Back 1S device emitting radiofrequency waves (see appendix). The principle is based on the application of a flat transducer in direct contact with the perineum which emits long high frequency waves (between 0.8MHz and 1.2MHz); a return pad will be placed under the patient to allow the waves to reach the target zone.

Sessions, lasting 15 minutes, will be carried out on d1 and on d2 postpartum by the midwife on duty for the patients included in the study, in a supine position with legs bent.

- - - 1. **Group B: No radiofrequency therapy**

After inclusion in group B, patients will be treated by applying a flat transducer in direct contact with the perineum without any emission of high frequency long waves in order to respect the blind aspect of the study. It should be noted that the patients in this group will not know that they are not receiving radiofrequency treatment. A return pad will also be placed under the patient to allow the waves to reach the target zone.

Sessions, lasting 15 minutes, will be carried out on d1 (RF1) and on d2 (RF2) postpartum by the midwife on duty for the patients included in the study, in a supine position with legs bent.

- 1. **Judgment criteria**
     - 1. **Primary judgment criterion**

The primary judgment criterion will be perineal pain.

The measurement of this criterion is detailed in section IV.E.3

Pain will be assessed:

- At the patient’s bedside prior to the first session (E1)
- At the patient’s bedside after the first session (E2)
- At the patient’s bedside prior to the second session (E3)
- At the patient’s bedside after the second session (E4)
- By phone interview on d15 postpartum (E5)
- During the clinical examination at 1 month postpartum (E6)

These assessments will be carried out by a research midwife.

- - - 1. **Secondary judgment criteria**

The secondary judgment criteria will be the following:

- The patient’s quality of life will be assessed prior to the first session (C1), after the first session (C2), after the second session (C3) ; by phone interview at d15 postpartum (C4), at 1 month postpartum (C5)
- The intake of analgesics during their stay on the maternity ward
- Time until sexual intercourse and intercourse quality by phone interview on d15 postpartum (RS1) and at 1 month postpartum (RS2)
- The quality of wound healing assessed at the patient’s bedside after the second session (Ci1); by phone interview on d15 postpartum (Ci2), at 1 month postpartum (Ci3)

These assessments will be carried out by a research midwife.

Measurement of these criteria are detailed in section IV.E.3

- - - 1. **Evaluation**

Pain will be measured using a tool: a visual analogue scale. ^[24]^

The patients’ quality of life will be measured using a specific questionnaire SF-12 ^[25]^

Time until sexual intercourse and intercourse quality will have specific items in the questionnaires.

The quality of wound healing will be assessed via items included in the evaluation questionnaires, we could not find a healing scale adapted to perineal healing.

Analgesic intake will be quantified via dedicated items in the evaluation questionnaires.

- 1. **Planned number of patients and justification**

The number of patients necessary was calculated on the basis of a study carried out in 2013 comparing the efficacy of lidocaine vs ropivacaïne for perineal infiltration post-episiotomy [9].

It is based, on the one hand, on 80% of patients having a VAS above 4 on d1 and, on the other hand, the hypothesis of a 40% pain decrease in the radiofrequency group: a minimum of 31 patients per group would be necessary with 90% power and 5% significance.

- 1. **Duration of participation**

Inclusion is planned to last for 6 months. Each patient will be monitored for 1 month. The total study duration is 7 months. The North Hospital maternity has an average of 1832 vaginal deliveries per year, the number of patients to be included will therefore easily be reached within the inclusion period. ^[26]^

1. **Diagram and course of the trial**
   1. **Task distribution**

The midwives who have been informed of recruitment modalities will be able to include patients.

This information will be given on an information sheet intended for midwives on the labour ward.

During inclusion by the midwife from the labour ward or the postnatal department, after having checked the inclusion criteria and having the informed consent form signed, the midwife will collect data from the patient’s medical record and from her interview.

The patient will be randomized into one of 2 groups using a pre-established randomization list, after having signed the informed consent form:

- if the patient is included in group A: “radiofrequency therapy”

- if the patient is included in group B: “no radiofrequency therapy”

Randomization will be carried out by the research midwife.

- 1. **Monitoring organisation**

Patients will have two radiotherapy sessions (RF1, RF2) on d1 then on d2 postpartum respectively.

Data collection, by the research midwife, will be carried out as follows:

- - - Concerning pain and quality of life: on d1 prior to and after the first session; on d2 prior to and after the second session; on d15 by phone interview; at 1 month during the clinical examination
    - Concerning wound healing: on d2 after the second session; at 1 month during the clinical examination
    - Concerning analgesic intake: on d1 prior to the first session; on d2 after the second session
    - Concerning sexual intercourse: on d15 and at 1 month by phone interview and during a consultation

1. **Data processing**
   1. **Data collection**
      - 1. **Organisation**

Inclusion data will be collected by the midwife in charge of inclusion.

Data on quality of life and pain scores will be recuperated via the questionnaires filled in by the research midwife who does not know which group the patient belongs to.

After it has been collected, data is recorded and anonymised in an Excel data collection table.

- - - 1. **Data collected upon inclusion (see appendix paragraph XII)**
- Data related to obstetric history: gravidity, parity
- Data related to the circumstances of the birth: date, spontaneous or instrumentally assisted expulsion, presence or absence of epidural analgesics, duration of expulsive efforts
- Data related to perineal lesions, types of tear and of episiotomy and their localisation; suturing techniques used; with or without perineal infiltration
  - - 1. **Data collected during the trial (see appendix paragraph XII)**
- Data related to pain: visual analogue scale
- Data related to the quality of life: questionnaire on interferences with day-to-day life (sitting, walking, sleeping, urinating, caring for the newborn)
- Data related to analgesic intake: dose, when, which analgesic
- Data related to time until sexual intercourse and intercourse quality: started again or not, pain during intercourse, apprehension or not, sensations
- Data related to wound healing: presence of bruising, hardening, oedema, redness, wound re-opening
  1. **Data entry**

Data will be collected by the research midwife.

It will be anonymised prior to being entered into an Excel-type data collection table.

- 1. **Statistical analyses of data**
     - 1. **Analysis populations**

Two populations understudy are the two groups made up for the trial:

- - - Group A: “Radiofrequency therapy”
    - Group B: “No radiofrequency therapy”
    1. **Description of the population, initial comparability between the groups**

Comparability between the 2 groups (“radiofrequency therapy” and “no radiofrequency therapy”) will be calculated for all of the variables available upon inclusion in order to ensure initial comparability of quantitative variables using chi-squared tests (or Fisher’s exact test), and of categorical variables using average comparison (or non-parametric Kruskal-Wallis tests). Statistical significance will be set at 5% for type I errors.

- - 1. **Analysis of the primary judgment criterion**

Data will be collected using a numeric scale and categorised for VAS above 4. A statistic Chi-squared percentage comparison will be used to compare pain scores.

- - 1. **Analysis of secondary judgment criteria**

Chi-squared tests will be used to compare pain scores after the second session, at d15 postpartum and at 1 month postpartum. To avoid alpha error inflation, generalised linear models (with the LOGIT function as the link function) may be used, taking into account the number of repeated measurements.

Analgesic intake during the stay on the maternity ward will be quantified using the average daily dose.

Time until sexual intercourse and intercourse quality will be quantified on d15 postpartum from a phone interview and at 1 month postpartum during a consultation.

The quality of wound healing will be assessed via the presence or absence of several items defined on d2 after the second radiofrequency session, and at 1 month during the consultation.

- 1. **Serious adverse event management**

No serious adverse event is expected for the radiofrequency sessions.

1. **Planned duration of the trial**

The total duration of the trial is 7 months, inclusion is carried out at the same time as the trial.

| **CALENDAR** |  |
| --- | --- |
| Submission to the CPP (French Ethics Committee) | September 2016 |
| Beginning of inclusion | November – December 2016 |
| End of inclusion | May – June 2017 |
| Data analysis and publications | July 2017 |

1. **Expected results – Perspectives**

**The results expected** are a significant reduction of pain scores during the first days postpartum for women with perineal lesions caused by vaginal delivery, whether instrumentally assisted or not.

In addition to an improvement in the quality of life of these patients over these first few days.

**Perspectives**: carry out a larger trial, including patients with only slight perineal lesions (scratches, vulvar oedema), use of routine radiofrequency therapy in the postnatal department.

1. **Vigilance of clinical trials**
   1. **Definition**
      - 1. **Adverse event**

Any untoward medical occurrence in the biomedical research subject whether this occurrence has a causal relationship or not with this trial or with the investigational medicinal product(s) on trial.

- - - 1. **Serious adverse event (SAE)**

A serious adverse event is any event that:

- results in death,
- or is life-threatening for the person who participates in the trial,
- or which results in serious temporary incapacity or a medically significant or persistent disability,
- or which causes hospitalisation or prolongation of existing hospitalisation,
- or which causes an anomaly or congenital malformation,
- or any other event which is not listed above but can be considered to be “potentially serious”
- is considered to be medically pertinent event according to the investigator,
- or even an event requiring a medical intervention to avoid progression towards one of the cases listed above.
  - - 1. **Unexpected adverse effect**

Any adverse effect of the experimental medicinal product for which the type, the severity or the progression does not match the information given on the reference document: Summary of Product Characteristics or Investigator’s Brochure.

- 1. **Investigator’s responsibilities**
     - 1. **Modalities of detection and collection of adverse events**

All adverse events must be investigated, reported and recorded, processed and evaluated from the first visit (inclusion d0) until the end of the trial and until they have been resolved. Adverse events are reported:

- during planned clinical, biological or other examinations and with a systematic interview by the investigator;
- by spontaneous notification from the participants, who shall be informed of the necessity of contacting the investigating physician in the case of any adverse event.

All adverse events will be noted on the adverse event report form in the clinical report form.

- - - 1. **Reporting SAE**

There are no expected serious adverse events for the application of radiofrequency during the postpartum period. Any adverse effects will be noted in the clinical report form.

The sponsor must evaluate the causal relationship between the serious adverse event and the investigational medicinal product and treatments linked to the trial.

He assesses whether the adverse event is expected or unexpected using the reference document (Summary of Product Characteristics).

He reports, within the regulatory delays, all of the serious and unexpected adverse effects to the EMA (Eudravigilance, European pharmacovigilance database), to the competent Health Authorities and to the Ethics Committees concerned and informs the investigators at a frequency adapted to the trial.

The regulatory report must be within a maximum delay of:

- 7 calendar days for unexpected, fatal or life-threatening serious adverse effects. In these cases, supplementary, pertinent information must be sought and transmitted within another 8-day delay.
- 15 calendar days for all other unexpected serious adverse effects. Likewise, supplementary, pertinent information must be sought and transmitted within another 8-day delay.

In the case of a blinded trial, in general, the sponsor reports the unexpected serious adverse effect to the competent Health Authorities and to the Ethics Committees concerned after having unblinded the investigational medicinal product.

- - - 1. **Evaluation of causality**

In the event of healing anomalies this will be accurately evaluated, effectively, during the postpartum period, events such as bruising, pain, constipation, urinary or perineal infections, and metrorrhagia are frequent. These events will be treated according to current learned society recommendations (2015) ^[27]^

- - - 1. **Quality Control**

Quality Insurance and Control, under the promoter’s responsibility, will be performed in compliance with Good Clinical Practice (decisions of 24 November 2006) in order to guarantee that the integrity of collected data is respected, to protect patients, to respect the protocol and current legislation throughout the entire inclusion phase and patient monitoring by a clinical research assistant mandated by the sponsor.

The type and frequency of monitoring will be defined according to the definition of the level of monitoring linked to patient risk and will depend on the number of patients included, on the inclusion frequency and on difficulties discovered during the trial (procedures validated by the quality work group for the promotion of FHF which decides on the level of monitoring according to the risk for the subject - OECD Recommendation on the Governance of Clinical Trials, December 2012).

For this trial, the level of monitoring is classified as “minimal” with a category B patient risk. This means that it is necessary to check consent forms and the presence of the participation statement in the source dossier. If one or more consent forms and the participation statement do not comply, dossiers will be monitored in a random manner.

1. **Legal and ethical aspects**

This trial will be carried out in compliance with the Declaration of Helsinski (World Medical Association) recalling ethical principles applicable to medical research on human beings, in compliance with the Guide of Good Clinical Practice which harmonises the conduct of human experimentation according to different legislations an international level. The prospective promoter for this project is represented by the Assistance Publique des Hôpitaux de Marseille (Marseille Public Hospitals). Regulatory watch will be carried out by the Promoter. He will submit the project to the regulatory bodies for prior approval. This project is within the framework of interventional biomedical research, within the meaning of article L.1121-1, applied to a product mentioned in article L.5311-1 of the Code de la Santé Publique (French Public Health Regulations)(medicinal product); it comes under the new regulatory framework which applies to research “organised and practised on human beings with the aim to develop biological and medical knowledge”, which is the Loi de Santé Publique (French Public Health Act) n°2004-806 of 9 August 2004 concerning public health policies and its implementing decrees of 27 August 2006, aiming to bring French legislation in line with European law. For this reason, it will also be submitted to a Comité de Protection des Personnes (French Ethics Committee) for their favourable opinion.

An information sheet will be handed to the patients and informed consent will be required. These will be drawn up in compliance with regulatory recommendations, namely recalling the trial objective, the advantages and the risks linked to the trial, the course of the trial and all of the legal provisions to which the patient is entitled. This trial will be conducted according to Good Clinical Practice representing a group of quality requirements in the ethical and scientific fields, which must be respected during the planning, implementation, conduct, monitoring, quality control, audit, data collection, analysis and expression of the results. Respect of this good clinical practice ensures the protection of rights, the safety and the protection of the people who participate in this trial and the preservation of their anonymity as well as the credibility (integrity, authenticity, verifiability) and accuracy of data and of these research results.

This protocol will not be altered or changed without the approval of all of the investigators. Any amendment to the trial protocol must be notified to the Ethics Committee (CPP) if the alterations modify the ethical or medical and scientific aspects of the trial. The investigators pledge to respect current legislative obligations and to conduct this study in compliance with Good Clinical Practice. The protocols and observation forms will be handed over by the CRA at the implementation visit to the centre. Information collected from patients will remain strictly confidential. It will be kept in paper form in a locked premises. It will be computerised and automatically processed. This computer processing will not permit the direct or indirect identification of the patients. Entire data may only be consulted by the principal investigator and the promoter’s representatives, or transmitted to the Authorised Health Authorities if necessary. This study will be declared to the Commission Nationale Informatiques et Libertés (CNIL; French national commission for the protection of personal data and individual liberties) in accordance with current legislation (Loi Informatiques et Libertés (French data protection act) of 6 January 1978, as amended by the Act of 1^st^ July 1994 and the Decree of 9 May 1995). The participant must be informed of the nature of the data processed, its finality, the identity of individual and legal entities who will be sent this data. He/she retains the right to access and rectify this data via the physician of his/her choice, as well as the right to object in accordance with European Directive 95/46/EC. In accordance with the law of 4 March 2002 relating to patients’ rights and the quality of the health system, the global results of the trial can be transmitted to the patients at their request, directly or via the physician of their choice.

1. **References**

[1] Alison J. Macarthur, MD, Colin Macarthur, MBChB, PhD. Incidence, severity, and determinants of perineal pain after vaginal delivery: A prospective cohort study. *American Journal of Obstetrics and Gynecology (2004) 191, 1199-204*

[2] Persico G, Vergani P, Cestaro C, Grandolfo M, Nespoli A. Assessment of postpartum perineal pain after vaginal delivery: prevalence, severity and determinants. A prospective observational study. *Minerva Gynecol, 2013 Dec; 65(6):669-78*

[3] Turmo M, Echevarria M, Rubio P, Almeida C. Development of chronic pain after episiotomy**.** *[Rev Esp Anestesiol Reanim.](http://www.ncbi.nlm.nih.gov/pubmed/25555717) 2015 Oct; 62(8):436-42.*

[4] HAS. « Douleur chronique : reconnaître le syndrome douloureux chronique, l’évaluer et orienter le patient » - *Consensus formalisé – December 2008 (Document in French)*

[5] Skovlund, G. Fyllingen, H. Landre and B.-I. Nesheim. Comparison of postpartum pain treatments using a sequential trial design. I. Paracetamol versus placebo. *Eur J Clin Pharmacol (1991) 40:343-347*

[6] Skovlund, G. Fyllingen, H. Landre and B.-I. Nesheim. Comparison of postpartum pain treatments using a sequential trial design. II. Naproxen versus paracetamol. *Eur J Clin Pharmacol (1991) 40:539-542*

[7] [Kamondetdecha R](http://www.ncbi.nlm.nih.gov/pubmed/?term=Kamondetdecha%20R%5BAuthor%5D&cauthor=true&cauthor_uid=18575278), [Tannirandorn Y](http://www.ncbi.nlm.nih.gov/pubmed/?term=Tannirandorn%20Y%5BAuthor%5D&cauthor=true&cauthor_uid=18575278). [J Ibuprofen versus acetaminophen for the relief of perineal pain after childbirth: a randomized controlled trial.](http://www.ncbi.nlm.nih.gov/pubmed/18575278) *[Med Assoc](http://www.ncbi.nlm.nih.gov/pubmed/18575278)**[Thai](http://www.ncbi.nlm.nih.gov/pubmed/18575278)*[.](http://www.ncbi.nlm.nih.gov/pubmed/18575278)*2008 Mar; 91(3):282-6.*

[8] Yildizhan R, Yildizhan B, Sahin S, Suer N. Comparison of the efficacy of diclofenac and indomethacin suppositories in treating perineal pain after episiotomy or laceration: a prospective, randomized, double-blind clinical trial. *Arch Gynecol Obstet (2009) 280:735–738*

[9] Gutton C, et al. Lidocaine versus ropivacaine for perineal infiltration post-episiotomy, Int J Gynecol Obstet (2013), http://dx.doi.org/j.ijgo.2013.01.028

[10] Schinkel, N, et al. Perineal infiltration with lidocaine 1%, ropivacaine 0.75%, or placebo for episiotomy repair in parturients who received epidural labor analgesia: a double-blind randomized study. *International Journal of Obstetric Anesthesia, Volume 19, Issue 3, p 239-297, July 2010*

[11] Zhou F, Wang XD, Li J, Huang GQ, Gao BX. Hyaluronidase for reducing perineal trauma. *Cochrane Database of Systematic Reviews 2014, Issue 2. Art. No.: CD010441.*

[12] Kettle C, Dowswell T, Ismail KMK. Continuous and interrupted suturing techniques for repair of episiotomy or second degree tears. *Cochrane Database of Systematic Reviews* 2012, Issue 11. Art.No:CD000947.

[13] G.P. Ganzit, L. Stefanini, G. S. (2009). TECAR® THERAPY in the treatment of acute and chronic pathologies in sports FMSI (Italian Sports Medicine Federation)-CONI Institute of Sports Medicine, Torino.

[14] Vall, J. and al. (2012). Radiofréquence Monopolaire capacitive résistive à 448kHz comme thérapie de rééducation dans la médecine du sport. Presentados En Las XXIII Jordanas de La Asociación Espanola de Baloncesto. (Document in French)

[15] Benitez, M. P. (2009). Tecartherapy in knee and spinal pathologies.

[16] E. Parolo, M. O. (2009). Hyperthermia through resistive and capacitive energy transfer in the treatment of acute and chronic musculoskeletal lesions.

[17] Inglés, F. and al. (2005). Caractère effectif de la radiofréquence monopolaire capacitive/résistive dans le traitement des cervicalgies. Indiba Clinical Trial. (Document in French)

[18] Terranova, A. and al. (2008). Radiofréquence monopolaire capacitive/résistive à 448khz pour le traitement post opératoire des fracture du fémur. Europa Medicophysica. (Document in French)

[19] Manley J. (2012) The Effectiveness of Manual Therapy on Chronic Pelvic Pain: An Evidence-Based Review, (University of California San Francisco Spring).

[20] Serra Llosa M.L, Martinez Bustello S. Fisiotherapy treatment of hypertonia in pelvic floor. *Revista* *Urodinámica* *Aplicada.* *2007*

[21] Pinto R. (2009) Radiofrequencia monopolar capacitiva resistiva de 448khz en el enveicimiento facial cutanéo medecina estética.

[22] Mohannad Hawamdeh. The effectiveness of Capacitive Resistive Diathermy (Tecartherapy®) in acute and chronic musculoskeletal lesions and pathologie. [*European Journal of Scientific Research*](https://www.researchgate.net/journal/1450-202X_European_Journal_of_Scientific_Research)*(Impact Factor: 0.74). 02/2014; Volume 118(3)*

[23] Michnowski, M., Bellaiche, L., Massiot, M., Rousseau, E., & La, I. (2013). Mesure des effets intra osseux de la radiofréquence à 448kHz en mode capacitif à l’IRM : étude préliminaire. *Kinésithérapie, la Revue ; [Volume 13, Issue 134](http://www.sciencedirect.com/science/journal/17790123/13/134), February 2013, Pages 38 (Document in French)*

[24] Bouvenot, G. (1993). Evaluation de la douleur: les méthodes. *Oueneau P., Ostermann G., Le médecin, le patient et sa douleur, APNET, Paris*, 53-59.(Document in French)

[25] [Desouky TF](http://www.ncbi.nlm.nih.gov/pubmed/?term=Desouky TF%5BAuthor%5D&cauthor=true&cauthor_uid=22829308" \t "_blank), [Mora PA](http://www.ncbi.nlm.nih.gov/pubmed/?term=Mora PA%5BAuthor%5D&cauthor=true&cauthor_uid=22829308" \t "_blank), [Howell EA](http://www.ncbi.nlm.nih.gov/pubmed/?term=Howell EA%5BAuthor%5D&cauthor=true&cauthor_uid=22829308" \t "_blank). Measurement invariance of the SF-12 across European-American, Latina, and African-American postpartum women. Quality of Life Research.  2013 Jun; 22(5):1135-44

[26] ScopeSanté affilié à l’HAS. CHU/APHM HOPITAL NORD (APHM) (2014), <http://www.scopesante.fr/fiches-detaillees/130780521/> (In French)

[27] CNGOF. Recommandations pour la pratique clinique (RPC) (2015), Directives qualité & autres référentiels, [http://www.cngof.asso.fr/D_PAGES/PURPC _00.HTM](http://www.cngof.asso.fr/D_PAGES/PURPC%20_00.HTM) consulté le 10.02.2016 (In French)

1. **Appendix**

**Inclusion questionnaire**

**INCLUSION QUESTIONNAIRE**

RADIOFREQUENCY AND POSTPARTUM PERINEAL PAIN TRIAL

**SUBJECT N° :…..**

**OBSTETRIC HISTORY:**

Gravidity: ..........

Parity: ..........

**DATA RELATED TO THE BIRTH:**

Date :................................ Hours of labour: .............

Expulsion : Spontaneous

Instrumentally assisted

Epidural analgesics: Yes

No

Length of expulsive efforts: ...........

**DATA RELATED TO PERINEAL LESIONS:**

Tears in case of tears, number: ..........

Episiotomy

Localisation: ............................... ................................

.............................. ...............................

Suturing techniques used: Individual sutures

Continuous sutures

Intradermal continuous

Perineal infiltration: Yes

No

**Questionnaire at the patient’s bedside**

**QUESTIONNAIRE AT THE PATIENT’S BEDSIDE**

RADIOFREQUENCY AND POSTPARTUM PERINEAL PAIN TRIAL

SUBJECT N°_._ . .

POST-PARTUM: **d1:** E1 E2 *(circle the answer)*

**d2:** E3

**1/ Visual Analogue Pain Score:** *(circle the answer)*

**0 1 2 3 4 5 6 7 8 9 10**

No pain Most pain imaginable

**2/ Questionnaire on interference with day-to-day activities:** pain and/or discomfort during these different activities *(circle the answer)*

Pain Discomfort

- Sitting: yes / no yes / no
- Walking: yes / no yes / no
- Urinating: yes / no yes / no
- Sleeping: yes / no yes / no
- Caring for newborn : yes / no yes / no

**3/ Have you taken other analgesics?-** (Doliprane^®^, Spasfon^®^, Profénid^®^, Bi-Profénid^®^, Acupan^®^, homeopathy, cold...) *(circle the answer)*

yes / no

If yes,

Which one(s).................

At what dosage...............

At what time.................

Did it relieve the pain ?...........

**4/ Wound healing** *(Tick the items present)*

Bruise(s) Oedema Hardening Redness

Wound re-opened

**Phone questionnaire**

**PHONE QUESTIONNAIRE/CONSULTATION**

RADIOFREQUENCY AND POSTPARTUM PERINEAL PAIN TRIAL

SUBJECT N°_._ . .

POST-PARTUM: **d15** *(circle the answer)*

**d30**

**1/ Visual Analogue Pain Score:** *(circle the answer)*

**0 1 2 3 4 5 6 7 8 9 10**

No pain Most pain

imaginable

**2/ Questionnaire on the quality of life :** *(circle the answer)*

- **On the whole, do you think your state of health is**: 1 Excellent 2 Very good 3 Good 4 Mediocre 5 Poor
- **Due to your current state of health, are you restricted:**
  - **in moderate physical efforts (moving a table, hoovering, …)?** 1 Yes, very restricted 2 Yes, slightly restricted 3 No, not at all restricted
  - **going up several flights of stairs?** 1 Yes, very restricted 2 Yes, slightly restricted 3 No, not at all restricted
  - **remaining seated for a long time during a meal with no pain?** 1 Yes, very restricted 2 Yes, slightly restricted 3 No, not at all restricted
  - **breast-feeding or giving your child a bottle while seated?** 1 Yes, very restricted 2 Yes, slightly restricted 3 No, not at all restricted
- **Over the last few weeks, and due to your physical health:**
  - **have you done less things than you would have liked?** 1 All of the time 2 Most of the time 3 Often 4 Sometimes 5 Never
  - **have you been restricted doing certain things?** 1 All of the time 2 Most of the time 3 Often 4 Sometimes 5 Never
- **Over the last few weeks, and due to your emotional state (like feeling sad, nervous or depressed):**
  - **have you done less things than you would have liked?**1 All of the time 2 Most of the time 3 Often 4 Sometimes 5 Never
  - **have you had any difficulty doing things with as much care and caution as usual?** 1 All of the time 2 Most of the time 3 Often 4 Sometimes 5 Never
- **Over the last few weeks, how much did physical pain restrict your work or your household activities?** 1 Not at all 2 A little bit 3 Average 4 A lot 5 Hugely
- **The following questions concern the way you have felt over the last few weeks. For each question give the most appropriate answer.**
  - **Have there been moments where you have felt calm and relaxed?** 1 All of the time 2 Most of the time 3 Often 4 Sometimes 5 Never
  - **Have there been moments where you have felt full of energy?** 1 All of the time 2 Most of the time 3 Often 4 Sometimes 5 Never
  - **Have there been moments where you have felt sad and down?** 1 All of the time 2 Most of the time 3 Often 4 Sometimes 5 Never
- **Over the last few weeks, have there been moments where your physical or emotional state have got in the way of your social life and your relationship with other people, your family, friends, acquaintances?** 1 All of the time 2 Most of the time 3 Often 4 Sometimes 5 Never
- **After how many days were you able to walk normally to take your child for a walk or to go for a quick shop?** …… Days

**3/ Have you taken other analgesics?- (Doliprane^®^, Spasfon^®^, Profénid^®^, Bi-Profénid^®^, Acupan^®^, homeopathy, cold...)** *( circle the answer)*

Yes / No

If yes,

Which one(s).................

At what dosage...............

At what time.................

Did it relieve the pain ?...........

**4/ Concerning sexual intercourse** *(**circle the answer)*

Restarted sexual intercourse : Yes If yes, when: ..........

No

Pain during intercourse : Yes

No

Apprehension: Yes

No

Sensations: ...........................................

**5/Wound healing** *(Tick the items present)*

Bruise(s) Oedema Hardening Redness

Wound re-opened
